# Early life and socio-economic determinants of dietary trajectories in infancy and early childhood – results from the HSHK birth cohort study

**DOI:** 10.1186/s12937-021-00731-3

**Published:** 2021-09-07

**Authors:** Narendar Manohar, Andrew Hayen, Loc Do, Jane Scott, Sameer Bhole, Amit Arora

**Affiliations:** 1 School of Health Sciences, Western Sydney University, Penrith, NSW 2751 Australia; 2 Health Equity Laboratory, Campbelltown, NSW 2560 Australia; 3 Australian College of Physical Education, Sydney Olympic Park, NSW 2127 Australia; 4 Faculty of Health, School of Public Health, University of Technology Sydney, Ultimo, NSW 2007 Australia; 5 Faculty of Health and Behavioural Sciences, School of Dentistry, University of Queensland, Brisbane, QLD 4072 Australia; 6 Australian Research Centre for Population Oral Health, University of Adelaide, Adelaide, SA 5005 Australia; 7 Discipline of Nutrition and Dietetics, School of Population Health, Curtin University, Perth, WA 6102 Australia; 8 Oral Health Services, Sydney Local Health District and Sydney Dental Hospital, Surry Hills, NSW 2010 Australia; 9 Faculty of Medicine and Health, Sydney Dental School, The University of Sydney, Surry Hills, NSW 2010 Australia; 10 Translational Health Research Institute, Western Sydney University, Campbelltown, NSW 2560 Australia; 11 Discipline of Child and Adolescent Health, Faculty of Medicine and Health, Sydney Medical School, Westmead, NSW 2145 Australia

**Keywords:** Diet, Food frequency questionnaire, Trajectories, Patterns, Social determinants of health, Socio-economic inequality, Healthy lifestyle, Preschool children

## Abstract

**Background:**

Early childhood is a period when dietary behaviours are established. This study aimed to examine the longitudinal intake of core and discretionary foods and identify early life and socio-economic factors influencing those intakes.

**Methods:**

Mother-infant dyads (*n* = 934) from the *Healthy Smiles Healthy Kids study*, an ongoing birth cohort study*,* were interviewed. The information on ‘weekly frequency of core and discretionary foods intake’ using a food frequency questionnaire was collected at 4 months, 8 months, 1 year, 2 years and 3 years age points. Group-based trajectory modelling analyses were performed to identify diet trajectories for ‘core’ and ‘discretionary’ foods respectively. A multinomial logistic regression was performed to identify the maternal and child-related predictors of resulting trajectories.

**Results:**

The intake of core and discretionary foods each showed distinct quadratic (*n* = 3) trajectories with age. Overall, core foods intake increased rapidly in the first year of life, followed by a decline after age two, whereas discretionary foods intake increased steadily across the five age points. Multiparity (Relative Risk (RR): 0.46, 95%CI: 0.27–0.77), non-English speaking ethnicity of mother (RR: 0.66, 95%CI: 0.47–0.91) and having a single mother (RR: 0.40, 95%CI: 0.18–0.85) were associated with low trajectories of core foods intake whereas older maternal age (RR: 1.05, 95%CI: 1.01–1.08) and longer breastfeeding duration (RR: 1.02, 95%CI: 1.00–1.03) were associated with higher trajectories of core foods intake. Also, multiparity (RR 2.63, 95%CI: 1.47–4.70), low maternal education (RR 3.01, 95%CI: 1.61–5.65), and socio-economic disadvantage (RR 2.69, 95%CI: 1.31–5.55) were associated with high trajectories of discretionary foods intake. Conversely, longer duration of breastfeeding (RR 0.99, 95%CI: 0.97–0.99), and timely introduction of complementary foods (RR 0.30, 95%CI: 0.15–0.61) had a protective effect against high discretionary foods consumption in infancy and early childhood.

**Conclusion:**

Children’s frequency of discretionary foods intake increases markedly as they transition from infancy to preschool age, and the trajectories of intake established during early childhood are strongly influenced by socio-demographic factors and infant feeding choices. Hence, there is a need for targeted strategies to improve nutrition in early childhood and ultimately prevent the incidence of chronic diseases in children.

**Supplementary Information:**

The online version contains supplementary material available at 10.1186/s12937-021-00731-3.

## Background

Infancy and early childhood is a period of rapid growth and development, coupled with evolving dietary requirements and physiological needs [1]. It is also a critical period during which susceptibility to many chronic diseases is established. An optimal diet in the early years is essential for a child’s growth and development [2]. Additionally, dietary habits in early childhood lay the foundation for lifelong dietary preferences [3], and contribute to several health conditions such as obesity, dental caries, diabetes and metabolic syndrome [4, 5]. Hence, adopting healthy dietary habits in early childhood and identifying population groups with sub-optimal dietary patterns early in life are important for preventing or at least delaying the incidence of chronic diseases [6]. Furthermore, understanding the complexity of dietary patterns and the factors influencing these patterns may help in defining which foods and/or diets are amenable to change and at what stage of life.

The transitional period from infancy to early childhood, accompanied by social and educational development is an important period for establishing dietary patterns that may continue into adulthood [7]. Furthermore, parental practices during this period can serve as a model of dietary behaviours for the next generation [8]. Much attention has been directed towards developing healthy diet practices in children, however more research needs to be undertaken to understand the long-term dietary patterns or dietary ‘trajectories’ in early childhood. The term ‘trajectories’ is defined as ‘groups of individuals following similar patterns of a behaviour or outcome of interest over time’ [9].

In recent years, dietary patterns in children have been examined using statistical approaches such as Principal Component Analysis (PCA), factor analysis and cluster analysis [10–14]. These techniques capture the whole diet in combination rather than individual food items and enable the identification of factors associated with dietary patterns of children. Studies using such approaches have shown that children from high socio-economic status (SES) tend to consume higher quality diets compared to children from lower SES. Furthermore, maternal factors such as young age, lower education, unemployment, lower household income, multiparity, and smoking are predictors for unhealthy dietary patterns in children [10–14].

Lately, an innovative statistical approach known as Group-Based Trajectory Modelling (GBTM) has gained attention in health and clinical sciences research. The GBTM identifies clusters of individuals who follow similar trajectories of health behaviours over time [15]. A recent study from Longitudinal Study of Australian Children (LSAC) used GBTM to derive and compare longitudinal dietary patterns in two cohorts of children [16]. However, to the best of our knowledge, no study has examined GBTM-derived dietary patterns of Australian children (birth–3 years) and their determinants. The objectives of this study are to:

1. Describe the longitudinal dietary trajectories of core and discretionary foods of Australian children from birth to age 3 years; and
2. Identify the maternal and child-related determinants of the observed trajectories.

## Methods

### Data and participants

This study used prospective data collected from 2009/10 up to 2013, from the Healthy Smiles Healthy Kids (HSHK) birth-cohort study [17] in South Western Sydney (SWS). This cohort study has been well-described in earlier publications [17, 18]. In summary, the study sample comprised of mothers who gave birth to live infants (with no known medical condition and no physical or intellectual disability which was likely to influence dietary behaviours, hygiene practices and physical activity), between October 2009 and February 2010, in public hospitals located within the Sydney and South Western Sydney Local Health Districts (formerly known as Sydney South West Area Health Service).

Mother-infant dyads (*n* = 1035) were recruited during their first post-natal visit (4 to 6 weeks postpartum) by Child and Family Health Nurses (CFHNs), who explained the study and obtained written consent for participation. For non-English speaking participants, interpreter services and written material in the native language of major ethnic groups (i.e., Vietnamese, Arabic, Hindi, Assyrian, Cambodian, Cantonese, Mandarin, Samoan) were provided.

### Data collection

At 8 weeks postpartum, the first (baseline) telephone interview was conducted to record information mainly on socio-demographic characteristics and infant-feeding practices including breastfeeding and use of formula at that age point. Subsequently, five follow-up telephone interviews were conducted at 4 months, 8 months, 1 year, 2 years, and 3 years age points. Considering early identification of infant feeding practices was one of the primary objectives of the HSHK birth cohort study, the first follow-up interview was undertaken at 4 months because this is a period of transition from breastfeeding to solid (complementary) foods and variations existed in the international and national infant feeding guidelines and practices of that time. For example, although the World Health Organization (WHO) [19] and Australian National Health and Medical Research Council (NHMRC) [20] recommended that infants should be exclusively breastfed for 6 months and that complementary foods be introduced thereafter. The majority of Australian infants (91.5%) receive complementary foods prior to 6 months and just over one third (35.3%) by 4 months of age [21]. The questionnaire used in this study was adapted from the Iowa Fluoride study [22], the NSW Child Health Questionnaire [23], the National Child Oral Health Survey [24], Perth Infant Feeding Studies (PIFS I and II) [25, 26], and the HSHK pilot study [27].

### Outcome measures

#### Dietary intake assessment

At every follow-up interview, information on the child’s current dietary habits in terms of consumption of 32 individual food and drink items during the preceding 7 days was obtained from the mother. At each interview, a short food frequency questionnaire (FFQ) was used (see Additional file 1) and mothers were asked an open-ended question “In the past 7 days how often was your baby/child fed each of the following foods and/or drinks?”. A numerical response was recorded to represent the number of times the specified food and/or drink was eaten in a week.

The 32 listed foods (see Additional file 1) were categorised into ‘core’ and ‘discretionary’ foods based on the Australian Dietary Guidelines [28, 29]. The same method was used to categorise the foods in a previously published research [30]. Core foods (*n* = 12 food items) comprised of five food sub-groups: dairy (e.g., milk, cheese, plain yoghurt) (*n* = 4 food items), grains (e.g., cereals, bread, rice) (*n* = 3 food items), fruits, vegetables, and meat and its alternatives (e.g., red meat, poultry, fish, and eggs) (*n* = 3 food items). The discretionary foods (*n* = 20 food items) were categorised into two sub-groups: foods high in saturated fats and/or salt (e.g., potato chips and savoury snacks) (*n* = 2 food items), and foods and drinks with added sugars (e.g., fruit juice, confectionary, biscuits, cakes, sugar-sweetened beverages (SSBs), sweetened yogurt, ice cream) (*n* = 18 food items). Fruit juices were considered discretionary (specifically sugar-based) because of their high total sugar and energy value [28].

Diet can be summarised in different ways, such as dietary patterns, indices, or scores [31]. Dietary scores sum the number or frequency of foods consumed during a specified time that are considered to be healthy or unhealthy [31]. These scores are intuitive and analytically simple therefore, were used to construct dietary trajectories. In this study, for dietary trajectory analyses across the five specified time-points, the seven mutually exclusive food groups were collapsed into two major food groups i.e., core (*n* = 12 food items) and discretionary (*n* = 20 food items). The frequency (continuous data) of each food in the five individual core food groups and two discretionary food groups were summed, and then the totals of each of the five individual core food groups were summed to give the ‘frequency of total core food group intake’ and then the total frequency of the two discretionary groups were summed to give the ‘total of the discretionary food group frequency’. Hence, individual dietary patterns were developed for the two major food groups and the seven individual food sub-groups respectively. The focus of the present study was primarily on the two major food groups i.e., core and discretionary foods.

#### Predictors

Several maternal and child factors considered to be potential determinants of healthy and unhealthy dietary patterns in children were derived from the literature [10–14] and investigated in the analyses. Maternal factors were: mother’s age at child’s birth (in years), marital status (single or married/living with partner), level of education (<Year 12, completed school, college, or university), employment status at 4 months postpartum (not working or working), mother’s country of birth (Australia, other English-speaking, or non-English-speaking), number of children in household (1, 2, or ≥ 3), and area-level socioeconomic status (SES) (deciles 9–10 = least disadvantaged, deciles 7–8 = low disadvantaged, deciles 5–6 = moderately disadvantaged, deciles 3–4 = highly disadvantaged, or deciles 1–2 = most disadvantaged). The SES was classified by census-based Australian Bureau of Statistics Index of Relative Socioeconomic Advantage and Disadvantage (IRSAD) [32] using the participants’ residential postcode. This composite index summarises information on social and economic resources of households and people living in specific postcodes. The national standardised mean is 1000 (±100), with higher scores denoting higher advantage [32]. Child factors were gender (male or female), duration of breastfeeding and age of introduction of complementary (solid) foods (< 17-weeks, 17–25-weeks, or ≥ 26-weeks).

### Statistical analyses

All statistical analyses were performed using Stata Statistical Software version 15.0 (StataCorp, College Station, TX, USA).

#### Participant characteristics

The characteristics of the study sample were summarised as means and standard deviations for continuous variables, and frequency and percentages for categorical variables.

#### Dietary trajectory analyses

For the first objective, GBTM using a plug-in (PROC TRAJ) in Stata was used to construct the dietary trajectories. The GBTM analyses were restricted to those study participants for whom the dietary data was available from at least three interview periods. For missing dietary data, one major advantage of GBTM is that it assumes that missing data are missing at random and adjusts the model so that missing data do not contribute to the sample size or analytical outcome.

The GBTM is based on finite mixture modelling for approximating unknown trajectories across population members. The GBTM identifies clusters of individuals with similar trajectories, and the model itself forms the trajectories based on the maximum-likelihood estimation using a general quasi-Newton method [33]. The Bayesian information criteria (BIC) [34] is often used to help decide the number of groups (model selection) that best represent the heterogeneity in the trajectories of the study sample. However, the BIC does not always clearly identify a preferred number of groups. Therefore, the objective of selection of number of groups (model selection) should not be maximisation of some statistical parameter; rather, it is to summarise the data features in as parsimonious manner as possible.

For study analyses, Poisson-based model was chosen because of the continuous distribution (count data) of the food frequency data at each time point. The GBTM analysis is a two-step process: (1) select the number of groups and (2) determine the order of the polynomial defining each group’s trajectory (i.e., zero-order, linear, cubic, quadratic). We fitted a series of 2- to 6-group models, testing zero-order, linear, cubic, and quadratic specifications for the trajectory shapes, until the best fitting model (which was parsimonious and analytically tractable) was established.

#### Predictors of trajectory group membership

For the second objective, two multinomial logistic regression analyses were performed to determine the associations between predictors (i.e., maternal and child factors) and trajectory group membership for ‘core’ and ‘discretionary’ foods groups, using Stata. Relative risk ratios (RR) were generated since the dependent variable (trajectories) was categorical with more than two groups. The trajectory representing the lowest consumption group was used as the reference category for each of the regression models. Significance level of 5% was used for the analysis.

### Ethics approval and participant consent

Ethics approval to conduct this study was given by the former Sydney South West Area Health Service – RPAH Zone (ID number X08–0115), Liverpool Hospital, University of Sydney and Western Sydney University. All participants signed a written consent form prior to study commencement.

## Results

### Participants’ characteristics

**Fig. 1:**
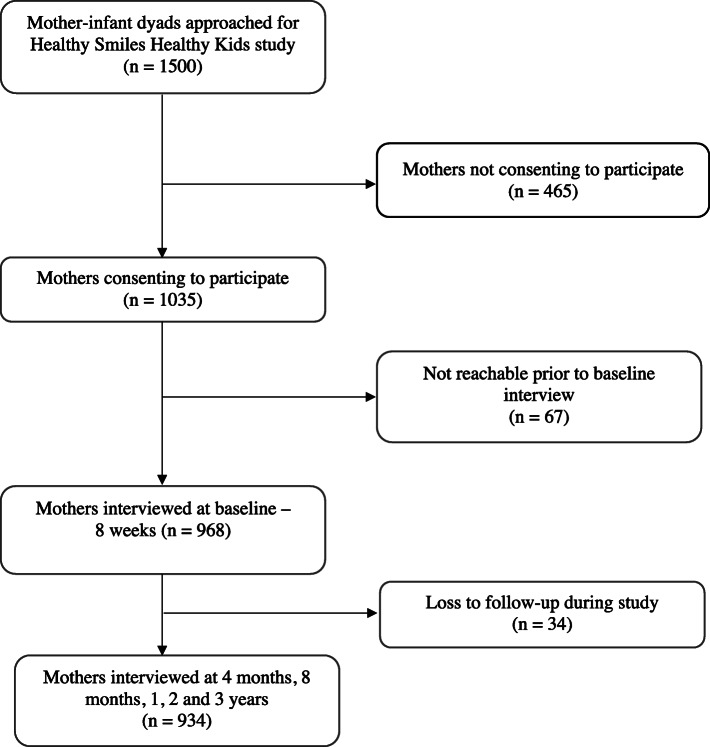
Flow chart of study sample recruitment and retention

A total of 1500 mothers were invited to participate in the HSHK study, of whom 1035 consented to participate (response rate - 69%). To ensure sample representativeness, socio-demographic characteristics and chosen method of infant feeding were compared between the participating and non-participating mothers (*n* = 465). There were no significant differences between the two groups in terms of maternal age (Chi-square (X^2^) = 4.75, *p* = 0.153), educational level (X^2^ = 6.65, *p* = 0.328), and method of infant feeding (X^2^ = 2.46, *p* = 0.813). Of the 1035, a further 67 mothers-infant dyads either opted out or were non-contactable (7 contact attempts made) before the baseline interview and 34 were lost to follow-up during the study period. In total, 934 participants had the dietary data (for at least three age points) required for this study (Fig. 1). Furthermore, 760 participants had complete dietary data for all five time points. There were no differences in the age, education level and method of infant feeding of mothers who completed interviews at 1 year, 2 years and 3 years, and those who withdrew from the study (data not reported). The characteristics of the mothers and their children are shown in Table 1.

**Table 1:** Characteristics of the study sample

Variables	Total Sample	Core Foods Trajectories ^**a**^			Discretionary Foods Trajectories ^**b**^		
		Group 1 (***n*** = 264)	Group 2 (***n*** = 426)	Group 3 (***n*** = 244)	Group 1 (***n*** = 364)	Group 2 (***n*** = 419)	Group 3 (***n*** = 151)
**Maternal factors**							
***Maternal age*** *(n = 933) Mean ± SD*	933 (31.22 ± 5.33)	264 (30.89 ± 5.18)	425 (30.86 ± 5.42)	244 (32.23 ± 5.22)	364 (31.63 ± 5.02)	418 (31.11 ± 5.19)	151 (30.58 ± 6.29)
***Maternal marital status****(n = 934)*							
Living with partner	844 (90.36%)	232 (27.49%)	378 (44.79%)	234 (27.73%)	344 (40.76%)	374 (44.31%)	126 (14.93%)
Single	90 (9.64%)	32 (35.56%)	48 (53.33%)	10 (11.11%)	20 (22.22%)	45 (50%)	25 (27.78%)
***Maternal education****(n = 934)*							
University	404 (43.25%)	101 (25%)	172 (42.57%)	131 (32.433%)	204 (50.5%)	158 (39.11%)	42 (10.4%)
College/TAFE	170 (18.20%)	40 (23.53)	99 (58.24%)	31 (18.24%)	71 (41.76%)	80 (47.06%)	19 11.18%)
Completed 12	192 (20.56%)	72 (37.5%)	78 (40.62%)	42 (21.88%)	51 (26.56%)	100 (52.08%)	41 21.35%)
Left school < 12	168 (18%)	51 (30.36%)	77 (45.83)	40 (23.81%)	38 (22.62%)	81 (48.21%)	49 29.17%)
***Employment status****(n = 932)*							
Not working	826 (88.63%)	231 (27.97%)	380 (46%)	215 (26.03%)	324 (39.23%)	368 (44.55%)	134 (16.22%)
Working	106 (11.37%)	32 (30.19%)	45 (42.45%)	29 (27.36%)	39 (36.79%)	50 (47.17%)	17 (16.04%)
***Maternal country of birth****(n = 934)*							
Australia-born	437 (46.79%)	109 (24.94%)	220 (50.34%)	108 (24.71%)	174 (39.82%)	197 (45.08%)	66 (15.1%)
English speaking country	60 (6.42%)	14 (23.33%)	32 (53.33%)	14 (23.33%)	26 (43.33%)	26 (43.33%)	8 (13.33%)
Non-English-speaking country	437 (46.79%)	141 (32.27%)	174 (39.82%)	122 (27.92%)	164 (37.533%)	196 (44.85%)	77 (17.62%)
***Number of children****(n = 934)*							
1	465 (49.79%)	116 (24.95%)	210 (45.16%)	139 (29.89%)	209 (44.95%)	192 (41.29%)	64 (13.766%)
2	283 (30.30%)	85 (30.04%)	131 (46.29%)	67 (23.67%)	111 (39.22%)	131 (46.29%)	41 (14.49%)
≥ 3	186 (19.91%)	63 (33.87%)	85 (45.7%)	38 (20.43%)	44 (23.66%)	96 (51.61%)	46 (24.73%)
***Index of relative socioeconomic advantage and disadvantage****(n = 934)*							
Deciles 9–10	221 (23.66%)	51 (23.08%)	104 (47.06%)	66 (29.86%)	113 (51.13%)	91 (41.18%)	17 (7.69%)
Deciles 7–8	160 (17.13%)	46 (28.75%)	66 (41.25%)	48 (30%)	74 (46.25%)	66 (41.25%)	20 (12.5%)
Deciles 5–6	30 (3.21%)	3 (10%)	22 (73.3%)	5 (16.67%)	11 (36.67%)	13 (43.33%)	6 (20%)
Deciles 3–4	220 (23.55%)	68 (30.91%)	106 (48.18%)	46 (20.91%)	74 (33.64%)	105 (47.73%)	41 (18.64%)
Deciles 1–2	303 (32.44%)	96 (31.68%)	128 (42.24%)	79 (26.07%)	92 (30.36%)	144 (47.52%)	67 (22.11%)
**Child factors**							
***Child gender*** *(n = 934)*							
Male	477 (51.07%)	133 (27.88%)	223 (46.75%)	121 (25.37%)	182 (38.16%)	205 (42.98%)	90 (18.87%)
Female	457 (48.93%)	131 (28.67%)	203 (44.42%)	123 (26.91%)	182 (39.82%)	214 (46.83%)	61 (13.35%)
***Duration of breastfeeding****(n = 932) Mean ± SD*	932 (29.56 ± 25.77)	264 (27.23 ± 27.37)	424 (28.29 ± 24.98)	244 (34.27 ± 24.81)	364 (34.36 ± 25.42)	417 (28.17 ± 26.07)	151 (21.84 ± 23.47)
***Age of introduction of solid foods*** *(n = 913)*							
< 17 weeks	111 (12.16%)	25 (22.52%)	72 (64.86%)	14 (12.61%)	27 (24.32%)	47 (42.34%)	37 (33.33%)
17-25 weeks	497 (54.44%)	136 (27.36%)	216 (43.46%)	145 (29.18%)	205 (41.25%)	216 (43.46%)	76 (15.29%)
≥ 26 weeks	305 (33.41%)	101 (33.11%)	120 (39.34%)	84 (27.54%)	130 (42.62%)	141 (46.23%)	34 (11.15%)

^a^ Core foods trajectories: Trajectory Group 1 - Lowest (Gradual increase with late decrease); Trajectory Group 2 - Medium (Rapid increase with late decrease); Trajectory Group 3 - Highest (Rapid increase with early decrease)

^b^ Discretionary foods trajectories: Trajectory Group 1 - Lowest (Low and gradual rising); Trajectory Group 2 - Medium (Moderate and gradual rising); Trajectory Group 3 - Highest (High and late declining)

Index of relative socioeconomic advantage and disadvantage: deciles 9–10 = least disadvantaged; deciles 7–8 = low disadvantaged; deciles 5–6 = moderately disadvantaged; Deciles 3–4 = highly disadvantaged and deciles 1–2 = most disadvantaged

a, b The total of the categories might not always add up to 934 due to missing or incomplete data for some items

N: sample size

### Dietary pattern trajectories

Trajectories were created for ‘core’ foods and ‘discretionary’ foods consumption patterns’ respectively. In terms of core foods trajectories, higher trajectories indicate a healthier diet whilst for discretionary foods trajectories, higher trajectories indicate an unhealthy diet. The individual trajectories for the seven mutually exclusive food groups (i.e., dairy, grains, fruits, vegetables, meat and its alternatives, foods with added fats and/or salt, and foods with added sugars) have been reported as Supplementary material (see Additional file 2).

### Trajectories of core foods and their predictors

**Fig. 2:**
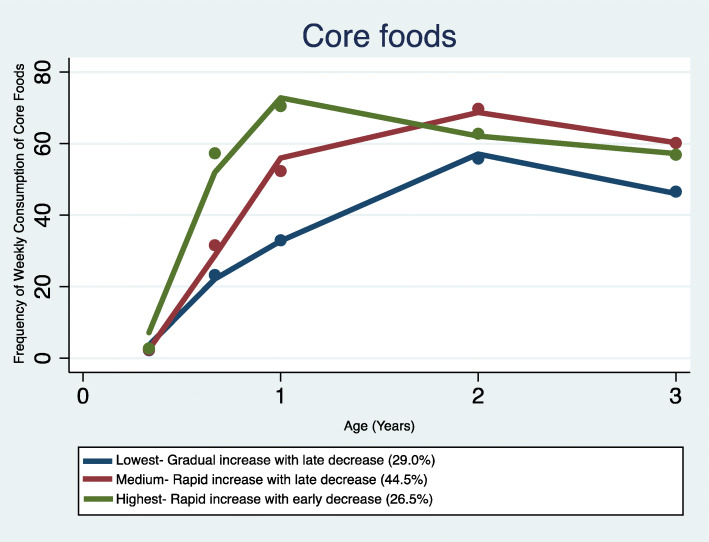
Trajectories of core foods consumption in infancy and early childhood

The GBTM identified three distinct core foods trajectories (Fig. 2): trajectory 1 (Lowest - gradual increase with late decrease) comprising of 29% of the sample; trajectory 2 (Medium - rapid increase with late decrease) comprising of 44.5%; and trajectory 3 (Highest - rapid increase with early decrease) comprising of 26.5% of the total sample. The obtained patterns suggest that increase in children’s core foods intake occurred between 4 months and 2 years of age, with frequency for all patterns decreasing between 2 and 3 years of age. From the age of 1 to 2 years, children with the highest consumption began to decrease their intake of core foods, while children in the lower consumption trajectories continued to increase their consumption until 2 to 3 years, after which a downward decline in core foods consumption was observed. Consequently, all three trajectories declined and converged with advancing age at the 3 years age point (Fig. 2). The distribution of sample characteristics by core foods trajectories are presented in Table 1.

#### Regression analyses

Table 2 shows the adjusted regression model of the association of maternal and child factors with core food trajectories during early childhood. After adjusting for covariates, compared with the reference trajectory 1 – ‘Lowest consumers - Gradual increase with late decrease’, children born to mothers who were born in non-English speaking country were less likely to follow trajectory 2 – ‘Medium consumers - Rapid increase with late decrease’ (RR: 0.66, 95%CI: 0.47–0.91; *p* = 0.013). Compared to the reference trajectory, children were less likely to follow trajectory 3 – ‘Highest consumers - Rapid increase with early decrease’ if they were born to a single mother (RR: 0.40, 95%CI: 0.18–0.85; *p* = 0.017) and or a mother with three or more children in their household (RR: 0.46, 95%CI: 0.27–0.77; *p* = 0.003), but were more likely to follow trajectory 3 if their mother was older (RR: 1.05, 95%CI: 1.01–1.08; *p* = 0.012) and breastfed longer (RR: 1.02, 95%CI: 1.00–1.03; *p* = 0.001).

**Table 2:** Factors associated with trajectories of core foods consumption in infancy and early childhood

Core food trajectories	Adjusted RR	95% CI		***P***
**Group 1**^**a**^	**(reference group)**			
**Group 2**^**b, d**^				
***Maternal country of birth***				
Australia-born	1.00			
English speaking country	1.24	0.63	2.44	0.535
Non-English-speaking country	0.66	0.47	0.91	0.013
**Group 3**^**c,e**^				
***Maternal age (in years)***	1.05	1.01	1.08	0.012
***Maternal marital status***				
Married	1.00			
Single	0.40	0.18	0.85	0.017
***Number of children***				
1	1.00			
2	0.66	0.43	1.00	0.052
≥ 3	0.46	0.27	0.77	0.003
***Breastfeeding duration (in months)***	1.02	1.00	1.03	0.001

*RR* Relative Risk Ratio, *95% CI* 95% Confidence Interval

^a^ Trajectory Group 1 - Lowest (Gradual increase with late decrease)

^b^ Trajectory Group 2 - Medium (Rapid increase with late decrease)

^**c**^ Trajectory Group 3 - Highest (Rapid increase with early decrease)

^d^ Adjusted for child gender, maternal age, maternal marital status, number of children, maternal education, maternal work status, index of relative socioeconomic advantage and disadvantage, age of introduction of solid foods, breastfeeding duration

^e^ Adjusted for child gender, maternal country of birth, maternal education, maternal work status, index of relative socioeconomic advantage and disadvantage, age of introduction of solid foods

### Trajectories of discretionary foods and their predictors

**Fig. 3:**
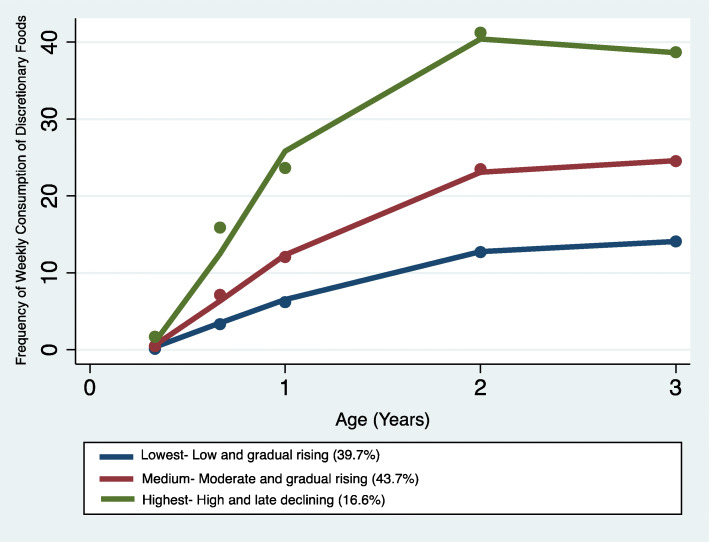
Trajectories of discretionary foods consumption in infancy and early childhood

Overall, the frequency of intake of discretionary foods in the study sample was high across all time points. The GBTM identified three distinct discretionary foods trajectories (Fig. 3): trajectory 1 – ‘Lowest consumers - Low and gradual rising’ comprising of 39.7% of the sample; trajectory 2 – ‘Medium consumers - Moderate and gradual rising’ comprising of 43.7%; and trajectory 3 – ‘Highest consumers - High and late declining’ comprising of 16.6% of the total sample. The obtained patterns suggest that children’s discretionary foods intake increased between 4 months and 2 years of age. Between the ages of 2 and 3 years, children who were initially the lowest consumers continued to have the lowest intakes, whilst children who had higher trajectories continued to maintain higher trajectories. Consequently, all the three trajectories remained distinctive with advancing age at the 3 years age point (Fig. 3). The distribution of sample characteristics by discretionary foods trajectories are also presented in Table 1.

#### Regression analyses

Table 3 shows the adjusted regression model of the association of maternal and child factors with discretionary foods trajectories during early childhood. After adjusting for covariates, compared with the reference trajectory 1 group - ‘Lowest consumers - Low and gradual rising’, children were more likely to follow trajectory 2 – ‘Medium consumers - Medium and gradual rising’ if they were born to mothers who had three or more children in household (RR: 1.97, 95%CI: 1.26–3.11; *p* = 0.003) and with low maternal education (RR: 1.81, 95%CI: 1.10–2.99; *p* = 0.019), whilst longer duration of breastfeeding reduced the risk of following trajectory 2 (RR: 0.99, 95%CI: 0.98–0.99; *p* = 0.029). Compared with the reference trajectory, being a girl (RR: 0.64, 95%CI: 0.42–0.97; *p* = 0.037), a longer duration of breastfeeding (RR: 0.99, 95%CI: 0.97–0.99; *p* = 0.029), and timely introduction of complementary foods (RR: 0.30, 95%CI: 0.15–0.61; *p* = 0.001) were associated with a lower risk of following trajectory 3 – ‘Highest consumers - High and late declining’. Conversely, having three or more children in the household (RR: 2.63, 95%CI: 1.47–4.70; *p* = 0.001), low maternal education (RR: 3.01, 95%CI: 1.61–5.65; *p* = 0.001), and being socio-economically disadvantaged (RR: 2.69, 95%CI: 1.31–5.55; *p* = 0.007) was associated with a higher risk of following trajectory 3.

**Table 3:** Factors associated with trajectories of discretionary foods consumption in infancy and early childhood

Discretionary food trajectories	Adjusted RR	95% CI		***P***
**Group 1**^**a**^	**(reference group)**			
**Group 2**^**b,d**^				
***Number of children***				
1	1.00			
2	1.15	0.82	1.62	0.407
≥ 3	1.97	1.26	3.11	0.003
***Maternal education***				
University	1.00			
College/TAFE	1.26	0.84	1.88	0.258
Completed 12	1.98	1.28	3.06	0.002
Left school < 12	1.81	1.10	2.99	0.019
***Breastfeeding duration (in months)***	0.99	0.98	0.99	0.029
**Group 3**^**c,e**^				
***Child gender***				
Male	1.00			
Female	0.64	0.42	0.97	0.037
***Number of children***				
1	1.00			
2	0.95	0.58	1.58	0.859
≥ 3	2.63	1.47	4.70	0.001
***Maternal education***				
University	1.00			
College/TAFE	1.01	0.53	1.94	0.962
Completed 12	2.18	1.20	3.94	0.010
Left school < 12	3.01	1.61	5.65	0.001
***Index of relative socioeconomic advantage and disadvantage***				
Deciles 9–10	1.00			
Deciles 7–8	1.67	0.78	3.599	0.185
Deciles 5–6	2.58	0.77	8.60	0.123
Deciles 3–4	2.59	1.26	5.29	0.009
Deciles 1–2	2.69	1.31	5.55	0.007
***Breastfeeding duration (in months)***	0.99	0.97	0.99	0.029
***Age of introduction of solid foods***				
< 17 weeks	1.00			
17-25 weeks	0.39	0.21	0.74	0.004
≥ 26 weeks	0.30	0.15	0.61	0.001

*RR* Relative Risk Ratio, *95% CI* 95% Confidence Interval

Index of relative socioeconomic advantage and disadvantage: deciles 9–10 = least disadvantaged; deciles 7–8 = low disadvantaged; deciles 5–6 = moderately disadvantaged; Deciles 3–4 = highly disadvantaged and deciles 1–2 = most disadvantaged

^a^ Trajectory Group 1 - Lowest (Low and gradual rising)

^b^ Trajectory Group 2 - Medium (Moderate and gradual rising)

^c^ Trajectory Group 3 - Highest (High and late declining)

^d^ Adjusted for child gender, maternal age, maternal marital status, maternal country of birth, maternal work status, index of relative socioeconomic advantage and disadvantage, age of introduction of solid foods

^e^ Adjusted for maternal age, maternal marital status, maternal country of birth, maternal work status

## Discussion

### Longitudinal dietary trajectories

In this study, consumption of discretionary foods commenced as early as 4 months of age, as reported in previous studies [30, 35, 36]. The frequency of discretionary foods consumption also continued to increase with advancing age (from 4 months to 3 years of age). Using data from the Melbourne Infant Feeding Activity and Nutrition Trial (InFANT) Program, Lioret et al. [35] observed that frequency of discretionary foods consumption amongst children doubled between 9 and 18 months of age. The LSAC study also observed consistent non-healthy dietary trajectories from the age of 2 years and onwards [16]. Similar tracking of unhealthy dietary patterns has been reported in international studies [37, 38]. Energy-dense and nutrient-poor discretionary foods contribute substantial ‘empty calories’ to the diets of young children [35] and may displace foods of better nutritional quality and/or value [39].

This study identified inconsistency in the dietary trajectories of healthy ‘core’ foods with advancing age, while unhealthy ‘discretionary’ foods trajectories remained relatively consistent. In contrast, previous studies have observed a consistency in both the healthy and unhealthy dietary patterns [12, 14, 16]. However, this study shows that children’s core foods consumption declined after the second year of life. This might possibly reflect the age period at which children gain more independence over their dietary choices and/or influence of their parents’ purchasing behaviour [13]. However, the FFQ used consisted of a relatively limited list of dietary items, and as children transitioned to the family diet, they may have eaten other core and discretionary foods that were not captured via the food list.

Frequent exposure to specific foods in early years is known to influence taste development and food preferences in later life [3]. As new food experiences in infancy influence the transition from a milk diet to a solid food diet, frequent exposure to healthy foods is likely to increase their consumption in later life [40]. Similarly, a high exposure to discretionary foods is likely to negatively influence dietary habits and food preferences in later years [41]. The study findings confirm that the period between 4 months and 2 years is a time of significant dietary transition, potentially having lifelong health implications.

### Predictors of infant and childhood dietary trajectories

Children with older mothers were likely to have higher core foods trajectory scores, whilst no association was found between maternal age and high discretionary foods trajectory scores. The Avon Longitudinal Study of Parents and Children (ALSPAC) found older maternal age to be positively associated with healthy dietary patterns [37]. Prior studies have reported poor-quality diets in families with younger mothers [10, 14], possibly because they tend to cook less [42], and older mothers might have better knowledge and experience in infant nutrition [43]. Maternal marital status was negatively associated with high core foods trajectory scores. Children of single mothers consumed core foods less frequently compared to those living with both parents, as reported in a prior study [44]. As single parents are likely to have a lower household income, they may choose foods that meet the child’s energy needs at a lower cost rather than expensive foods of greater variety and health rating. Single parents may experience time constraints in preparing meals and resort to the use of less nutritious convenience foods [44]. Although maternal country of birth was found to be associated with lower core foods trajectory scores, no association was found between maternal country of birth and discretionary foods trajectories. Children of non-English-speaking mothers tended to consume core foods less frequently than those with Australian-born mothers, as reported in an Australian cohort study [45]. Ethnic differences in the dietary patterns are often reported [46], which signifies the influence of culture on dietary practices.

Low level of maternal education and belonging to the most socially disadvantaged quintile were strongly associated with children following the highest discretionary foods trajectories. Previous studies have identified parental education and socio-economic position as key determinants of unhealthy dietary patterns [14, 47]. Mothers’ role in children’s dietary behaviours is particularly important because they usually spend more time with their child and are more closely engaged in direct feeding interactions with their child [48]. Low educated mothers may have poor food literacy, and this is reflected in their own personal dietary choices and subsequently in their child’s diet [10]. The higher frequency of discretionary foods intake among children from the most socially disadvantaged quintile could be attributed to the cheaper prices of energy-dense and nutritious-poor foods [46]. Higher socio-economic status is also associated with greater food expenditure, which in turn is associated with healthier food purchasing [49].

Duration of breastfeeding and complementary feeding practices were observed to be strongly associated with dietary trajectories. As reported in earlier studies [10, 47], longer breastfeeding duration was associated with high core foods trajectories. Children who were introduced to solid foods very early (before 17 weeks of age) were most likely to have high trajectories of discretionary foods consumption. This may be explained by the effect of early feeding experiences on food and taste acceptance in later years [3]. Earlier introduction of solid foods is also associated with early introduction of discretionary foods (before 52 weeks of age) and a greater preference for discretionary foods [50]. These findings suggest that mothers who introduce solid foods early may also introduce discretionary foods early. Shorter breastfeeding duration has also been associated with a greater consumption of discretionary foods [50]. Longer breastfeeding duration provides ongoing exposure to a variety flavours not experienced by formula-fed infants [51], and positively influences children’s vegetable intake [52]. These findings suggest that educating first-time and young mothers about the importance of breastfeeding and timely introduction of complementary foods is likely to improve their child’s long-term dietary habits.

Boys were found to be higher consumers of discretionary foods compared to girls. Previous studies have also documented that boys exhibited poor dietary patterns, and consumed higher amounts of processed starches, bread, pastry, chips, fast-food and sugar-sweetened beverages [11, 14]. As boys’ energy requirements are higher than girls, their greater liking for energy-dense food groups might be an adaptive response [53]. Furthermore, boys have been reported to have a greater liking for fatty and sugary foods, whereas girls are likely to prefer fruits and vegetables [53]. Similarly, multiparity was found to be associated with lower consumption of core foods and higher consumption of discretionary foods, thus confirming the negative influence of siblings on diet quality as reported in other studies [14, 47]. Having three or more children makes it difficult for the mother to prepare adequate meals as they are busy caring for their children. Parents may also introduce discretionary foods to younger children that are typically given to older siblings, or the older siblings may share such foods with their younger siblings [54].

### Implications for practitioners or policymakers

As non-healthy dietary trajectories were observed from as early as 4 months of age, it is important to target interventions in the antenatal and postpartum periods. The deterioration in the consumption of core foods from the age of 2 years might indicate that as children grow older, they develop a preference for discretionary foods; they are negatively influenced by older siblings; or they may persuade parents to purchase unhealthy foods. Children, particularly boys, breastfed for shorter duration and given solid foods very early, with two or more older siblings; born to less educated, and socioeconomically disadvantaged mothers are more likely to have high discretionary foods trajectories. Understanding of the factors that influence healthy and unhealthy dietary patterns can assist policy makers and health professionals, including general practitioners and mid-wives to design and provide more targeted and culturally appropriate support and more practical advice to the families, especially mothers (as being primary caregiver) as early as the pre-pregnancy counselling stage. Such early interventions will help to improve the dietary patterns through better compliance with the Australian infant feeding guidelines.

Additionally, the identified associations between healthy and unhealthy diet trajectories with breastfeeding duration and introduction to solids respectively can be used to inform the NSW First 2000 days Framework [55] and breastfeeding policies [56]. Furthermore, the study findings will inform policy makers on the inclusion of specific types and frequency of foods that should be eaten by children and more practical tips for parents [45] in the future Australian Dietary Guidelines planned to be released in 2024. Findings will also inform the South Western Sydney Local Health Districts’ Growing Healthy Kids in SWS strategy [57] which aims to increase children’s preference to core foods over discretionary foods by creating healthy food environments.

### Strengths and limitations

One of the major strengths was that same instrument was used to record children’s diet at evenly spaced time intervals over a three-year period. The repeated recording provides a reliable representation of longitudinal dietary exposures. The frequency of dietary intake was recorded for 7 days prior to the interview, which may better represent a child’s habitual intake. This study used an innovative GBTM analysis, which enables identification of heterogeneity in the development of ‘core’ and ‘discretionary’ foods dietary patterns and avoids the use of subjective criterion and cut-offs for identification of consumer groups. Furthermore, this study explores the frequency of core and discretionary foods consumption in first 3 years of life whereas, earlier Australian studies only assessed the dietary patterns in older children or adults [13, 16]. A large number of participants were retained (*n* = 934) and included in the trajectory analyses, providing precision and power. Finally, inclusion of a wide range of maternal and child measures assisted in identifying potential intervention strategies.

In term of limitations, children’s dietary intake was parent-reported, so there may be a possibility of underreporting, social desirability bias and/or inaccurate dietary recall. However, data collection at regular time intervals minimised the chances of heaping of data and recall bias. Although the FFQ was adapted from well-established literature, it is unlikely to have captured the whole diet and some items were not recorded at every follow up interview. Furthermore, its validity and appropriateness for the cultural and linguistically diverse participants need to be considered. However, FFQ are easy to administer, and cost-effective therefore, commonly used in large and/or longitudinal studies [58]. Hence, it assisted in maintaining good retention in the present study. Furthermore, at each age interval, the dietary measures attempted to cover most of the essential core and discretionary foods listed in the Australian dietary guidelines however, the number of items listed were limited (i.e., 12 items for core foods and 20 for discretionary foods) and some items were not recorded at every follow up interview. The inclusion of extra dietary items/questions might have produced more or different dietary trajectories. Additionally, only the frequency of dietary intake was recorded rather than both frequency and serving size, so we could not capture actual intake since the serving size might vary within and between individuals with advancing age. Within core foods group, refined cereals could not be distinguished from unrefined cereals, therefore, some cereals might have been included that are high in sugar [58]. Lastly, the purpose of group-based trajectory modelling is to establish a statistical approximation of a more complex reality by creating ‘trajectory’ groups based on analytical convenience. However, the trajectory groups do not exist in real sense. Furthermore, the PROC TRAJ plug-in (used in this study) does not take into account the growth factor variances within the trajectory classes, which theoretically may affect trajectory group membership of some cases. However, such variation is unlikely to affect the overall outcome analysis and associated inferences [59]. Lastly, like every statistical method, GBTM has its limitations [60]. Nonetheless, GBTM seems to be a valid and effective tool to investigate the patterns (or trajectories) of dietary intake across the life-course.

## Conclusion

The dietary trajectories in this study sample indicate an important risk of nutritional inadequacy for some children, with a decline in core foods intake being observed from the age of 2 years. Moreover, consumption of energy-dense, nutrient poor discretionary foods commenced as early as 4 months of age and steadily increased thereafter. These findings have important public health implications, since the behaviours influencing these dietary trajectories can be modified. The early childhood diet trajectories observed in this study are influenced by maternal socio-demographic characteristics, family size and maternal feeding practices. This study provides important evidence for promoting healthy dietary trajectories in infants, with the involvement of parents.

## Supplementary Information

**Additional file 1: Table S1.** List of dietary items (*n* = 32) recorded in the Short Food Frequency Questionnaire.

**Additional file 2.** Graphs depicting the individual trajectories of seven mutually exclusive food groups.

## Data Availability

The data of this study can’t be shared publicly due to the presence of sensitive (confidential) participants’ information.

## Abbreviations

ALSPAC: Avon Longitudinal Study of Parents and Children
BIC: Bayesian information criteria, BIC
IRSAD: Census Index of Relative Socioeconomic Advantage and Disadvantage
CFHNs: Child and Family Health Nurses
CI: Confidence interval
FFQ: Food frequency questionnaire
GBTM: Group-based trajectory modelling
HSHK: Healthy Smiles Healthy Kids
InFANT: Infant Feeding Activity and Nutrition Trial
LSAC: Longitudinal Study of Australian Children
PIFS: Perth Infant Feeding Studies
PCA: Principal component analysis
RRR: Relative risk ratios
SES: Socio-economic status
SSBs: Sugar-sweetened beverages
