# Supplementary material for: Early life and socio-economic determinants of dietary trajectories in infancy and early childhood – results from the HSHK birth cohort study
Source: Nutr J. 2021 Sep 7;20:76. doi: 10.1186/s12937-021-00731-3 (PMC8424821; doi:10.1186/s12937-021-00731-3)
Supplement: Supplementary file 1 — Additional file 1: Table S1. List of dietary items (n = 32) recorded in the Short Food Frequency Questionnaire. [file 12937_2021_731_MOESM1_ESM.docx]

**Additional file 1 – Table 1: List of dietary items (n = 32) recorded in the Short Food Frequency Questionnaire:**

|  | **Interview Phases** | | | | |
| --- | --- | --- | --- | --- | --- |
| **Dietary Items (**In the past 7 days how often (count data) was your baby/child fed each of the following foods and/or drinks?) | **4-months** | **8-months** | **1-year** | **2-years** | **3-years** |
| ***Core (n = 12)*** |  |  |  |  |  |
| **Dairy** |  |  |  |  |  |
| Cow milk |  |  |  |  |  |
| Other milk: soy milk, goat milk, rice milk |  |  |  |  |  |
| Plain Yoghurt |  |  |  |  |  |
| Cheese |  |  |  |  |  |
| **Grains** |  |  |  |  |  |
| Baby cereal |  |  |  |  |  |
| Cereals |  |  |  |  |  |
| Other starches (e.g., breakfast cereals, bread, rice, pasta, crackers) |  |  |  |  |  |
| **Fruits** |  |  |  |  |  |
| **Vegetables** |  |  |  |  |  |
| **Meat and alternatives** |  |  |  |  |  |
| Meat, chicken, combination dinners |  |  |  |  |  |
| Fish or shellfish |  |  |  |  |  |
| Eggs |  |  |  |  |  |
| ***Discretionary (n = 20)*** |  |  |  |  |  |
| **Foods with added sugars** |  |  |  |  |  |
| Flavored milk (e.g., Milo™, Nesquik™, Chocolate milk) |  |  |  |  |  |
| Flavored yogurt |  |  |  |  |  |
| Ice cream, custard and other dairy desserts |  |  |  |  |  |
| Fruit Juice (if diluted, state the %) |  |  |  |  |  |
| Soft drinks (e.g., Coke™) |  |  |  |  |  |
| Cordial including Ribena™ |  |  |  |  |  |
| Sports drinks (e.g., PowerAde™ and Gotarade™) |  |  |  |  |  |
| Powdered drink (e.g., Tang™) |  |  |  |  |  |
| Flavored mineral water |  |  |  |  |  |
| Iced Tea |  |  |  |  |  |
| Iced Coffee |  |  |  |  |  |
| Hot Tea (if sugar added) | X | X | X |  |  |
| Hot Coffee (if sugar added) | X | X | X |  |  |
| Lollies (candy), chocolate and sugar-based confectionary |  |  |  |  |  |
| Syrups, jams and sweet spreads (e.g., Nutella, honey and maple syrup) |  |  |  |  |  |
| Honey |  |  |  | X | X |
| Packaged sweet snacks e.g., muesli bars/fruit straps | X | X | X |  |  |
| Biscuits, cakes and/or puddings |  |  |  |  |  |
| **Foods with added fats and/or salt** |  |  |  |  |  |
| French fries/ hot chips |  |  |  |  |  |
| Packed snacks such as Potato chips/crisps | X | X | X |  |  |

**Note:** X: Frequency of intake NOT recorded at that specific interview phase
