# Supplementary material for: Early life and socio-economic determinants of dietary trajectories in infancy and early childhood – results from the HSHK birth cohort study
Source: Nutr J. 2021 Sep 7;20:76. doi: 10.1186/s12937-021-00731-3 (PMC8424821; doi:10.1186/s12937-021-00731-3)
Supplement: Supplementary file 2 — Additional file 2. Graphs depicting the individual trajectories of seven mutually exclusive food groups. [file 12937_2021_731_MOESM2_ESM.docx]

**Additional file 2 – Graphs depicting the trajectories of seven food groups**

**PART I: TRAJECTORIES OF INDIVIDUAL ‘CORE’ FOODS GROUPS**

**Figure 1: Trajectories of dairy consumption in infancy and early childhood**

**Figure 2: Trajectories of grains consumption in infancy and early childhood**

**Figure 3: Trajectories of fruits consumption in infancy and early childhood**

**Figure 4: Trajectories of vegetables consumption in infancy and early childhood**

**Figure 5: Trajectories of meat and its alternatives consumption in infancy and early childhood**

**Note:**

* This group includes red meat, chicken, fish and eggs

**PART II: TRAJECTORIES OF INDIVIDUAL ‘DISCRETIONARY’ FOODS GROUPS**

**Figure 6: Trajectories of fat and/or salt-based foods consumption in infancy and early childhood**

**Figure 7: Trajectories of sugar-based foods consumption in infancy and early childhood**
